# Prodromal Alzheimer’s Disease: Constitutive Upregulation of Neuroglobin Prevents the Initiation of Alzheimer’s Pathology

**DOI:** 10.3389/fnins.2020.562581

**Published:** 2020-12-03

**Authors:** Silvia de Vidania, Irene Palomares-Perez, Ana Frank-García, Takashi Saito, Takaomi C. Saido, Jonathan Draffin, María Szaruga, Lucía Chávez-Gutierrez, Miguel Calero, Miguel Medina, Francesc X. Guix, Carlos G. Dotti

**Affiliations:** ^1^Molecular Neuropathology, Physiological and Pathological Processes, Centro de Biología Molecular Severo Ochoa, CSIC/UAM, Madrid, Spain; ^2^Department of Neurology, Instituto de Salud Carlos III (ISCIII), Division Neurodegenerative Disease, University Hospital La Paz, Madrid, Spain; ^3^Laboratory for Proteolytic Neuroscience, RIKEN Center for Brain Science, Wako-shi, Japan; ^4^Department of Neurocognitive Science, Nagoya City University Graduate School of Medical Science, Nagoya, Japan; ^5^KU Leuven Department for Neurosciences, VIB-KU Leuven Center for Brain and Disease Research, Leuven, Belgium; ^6^CIBERNED, Queen Sofia Foundation Alzheimer Center, CIEN Foundation, Instituto de Salud Carlos III, Madrid, Spain

**Keywords:** Alzheimer’s disease, Amyloid-beta peptide, dendritic complexity, neuroglobin, resilience

## Abstract

In humans, a considerable number of the autopsy samples of cognitively normal individuals aged between 57 and 102 years have revealed the presence of amyloid plaques, one of the typical signs of AD, indicating that many of us use mechanisms that defend ourselves from the toxic consequences of Aß. The human APP NL/F (hAPP NL/F) knockin mouse appears as the ideal mouse model to identify these mechanisms, since they have high Aß42 levels at an early age and moderate signs of disease when old. Here we show that in these mice, the brain levels of the hemoprotein Neuroglobin (Ngb) increase with age, in parallel with the increase in Aß42. *In vitro*, in wild type neurons, exogenous Aß increases the expression of Ngb and Ngb over-expression prevents Aß toxicity. *In vivo*, in old hAPP NL/F mice, Ngb knockdown leads to dendritic tree simplification, an early sign of Alzheimer’s disease. These results could indicate that Alzheimer’s symptoms may start developing at the time when defense mechanisms start wearing out. In agreement, analysis of plasma Ngb levels in aged individuals revealed decreased levels in those whose cognitive abilities worsened during a 5-year longitudinal follow-up period.

## Introduction

Numerous studies in different experimental settings demonstrate the direct association between an excess of oligomeric forms of Aβ peptide and the appearance of some of the pathological signs characteristics of Alzheimer’s disease (AD) (Walsh et al., 2002; Li et al., 2009; Dinamarca et al., 2012). However, 30 to 50% of cognitively normal individuals aged between 57 and 102 years show AD pathological hallmarks after autopsy (Haroutunian et al., 1999; Aizenstein et al., 2008). This suggests that environmental and biological factors may offer compensation against AD pathological hallmarks. The proof *in vivo* that it is possible to counteract the toxicity exerted by a high Aβ burden by biological factors is found in a particular group of early-onset AD patients carrying the presenilin 1 (PSEN1) E280A mutation that increase Aβ production and induce amyloid accumulation in the brain. While PSEN1-E280A carriers develop dementia at the median age of 49 years, carrying a mutation on the AD risk modifier ApoE3 (R136S) delayed dementia significantly, until the 7th decade (Arboleda-Velasquez et al., 2019), demonstrating that Aβ accumulation is not enough to develop AD. By impairing the binding to heparan sulfate proteoglycans (HSPGs), the ApoE3 (R136S) mutant may avoid tau pathology spread (Arboleda-Velasquez et al., 2019) and Aβ – induced microglia activation in the brain (Bussini et al., 2005). Even more, there is a time-window during which the presence of oligomeric forms of this peptide does not produce significant alterations, implying the action of mechanisms that counteract the toxic effects of these peptides. As a matter of fact, data accumulated over the last years have revealed that neuropathological and biochemical changes occur decades before neuro-degeneration and clinical manifestation of AD symptoms (Troncoso et al., 1996, 1998; Price and Morris, 1999; Bennett et al., 2006). The study of autosomal dominant forms of the disease have revealed that Aβ deposition in the brain of pre-symptomatic individuals is observed up to 20 years before the onset of the symptoms (Rodriguez-Vieitez et al., 2016). Also, in sporadic forms of AD, increased Aβ levels are detected in plasma-derived neuronal exosomes up to 10 years before the manifestation of the first symptoms (Fiandaca et al., 2015). All these data imply the existence of robust resilience mechanisms, such as the recently identified PLA2 G4E (Perez-Gonzalez et al., 2020), counteracting the detrimental effect of different toxicity factors, including excessive production of the more damaging forms of Aβ peptide. The identification of these mechanisms is a fundamental process if we want to prevent or cure this disease.

There have been numerous proposals for mechanisms that protect neurons against the toxicity of Aβ aggregation, effective in *in vitro* and *in vivo* models (Yan and Wang, 2008). However, these works highlight *potential mechanisms* that could protect but do not help us to know what *physiological* mechanisms are operating to protect the brain of subjects exposed during a long period of time to elevated levels of the toxic forms of Aβ. A good model to look for physiological constitutive protective mechanisms is the humanized (Aβ region) APP knockin mouse bearing the Swedish and Iberian mutations (hAPP NL/F), since this mouse presents high levels of Aβ42 from very early ages with mild symptoms of pathology at late ages (Saito et al., 2014; Masuda et al., 2016).

## Materials and Methods

### Antibodies

The following antibodies were used for the Western Blot analysis: rabbit anti-Akt (Cell Signaling, dilution 1:500), rabbit anti-pSer473 Akt (Cell Signaling, dilution 1:500), rabbit anti-Bcl-2 (Bioworld, dilution 1:500), rabbit anti-Ubiquitin (Santa Cruz, dilution 1:500), mouse anti-Tau 5 (Thermo, dilution 1:500), mouse anti-GAPDH (Abcam, dilution 1:10,000), rabbit anti-GSK3β (Invitrogen, dilution 1:500), rabbit anti-Hsp-70 (Enzo, dilution 1:1000), rabbit anti-JNK (Cell Signaling, dilution 1:500), rabbit anti-LAMP 2A (Invitrogen, dilution 1:500), rabbit anti-LC3 (Sigma, dilution 1:1000), rabbit anti-Ngb (Genetex, dilution 1:500), rabbit anti-P62 (Cell signaling, dilution 1:500), rabbit anti-pThr180/Tyr182 p38 MAPK (Cell Signaling, dilution 1:1000), rabbit anti-pThr183/Tyr185 JNK (Cell Signaling, dilution 1:500), mouse anti-pTyr216 GSK3β (Millipore, dilution 1:500), rabbit anti-p38 MAPK (Abcam, dilution 1:1000) and rabbit anti-Ser396/Ser404 Tau (PHF1) (provided by Prof. Jesus Ávila, CBMSO, Spain, dilution 1:500). For the immunofluorescence analysis the following antibodies were used: mouse anti-DDK (FLAG) (Origene, dilution 1:1000), rabbit anti-CD45 (Abcam, dilution 1:1000), rabbit anti-GFAP (Millipore, dilution 1:1000), rabbit anti-Iba-1 (Wako, dilution 1:1000), rabbit anti-NeuN (Abcam, dilution 1:1000), rabbit anti-Ngb (Sigma-Aldrich, dilution 1:200) and rabbit anti-tGFP (Thermo, dilution 1:2500).

### hAPP NL/F Mice

hAPP NL/F mice were made on C57BL/6J background as detailed in Saito et al. (2014). All the animals were kept in the Centro de Biología Molecular Severo Ochoa’s (CBMSO) animal facility. The mice and manipulations presented in this work count with the approval of the Dirección General de Medio Ambiente de la Comunidad Autónoma de Madrid (Ref. PROEX 066/15) and the CBMSO’s Ethical Committee. All the experiments were performed in accordance with European Union guidelines (2010/63/UE) regarding the use of laboratory animals. Only males were used for this study, the ages of which are specified in each figure legend. All the experimental procedures with laboratory animals were carried out in accordance with the European directive transposed into the Spanish legal system in RD 53/2013, of 1 February, which establishes the basic rules applicable for the protection of animals used in experimentation and other scientific purposes.

### Genotyping

For the genetic identification of homozygous hAPP NL/F mice, genomic DNA was extracted from the tail by digestion of the tissue with proteinase K (Merck) at 10 mg/ml in lysis buffer (50 mM Tris–HCl pH 8, 100 mM NaCl, 100 mM EDTA, SDS 1%) at 60°C for 2 h and then the DNA was purified to carry out the polymerase chain reaction (PCR). The fragments corresponding to the WT allele (700 bp) or the mutated allele (400 bp) were amplified using the following oligonucleotides: WT forward ATCTCGGAAGTGAAGATG, WT reverse TGTAGATGAGAACTTAAC; NL-F forward AT CTCGGAAGTGAATCTA, NL-F reverse CGTATAATGTATG CTATACGAAG. A PCR program with the following protocol (T^a^, duration, number of cycles) was used: (i) 94°C, 3 min, 1 cycle; (ii) 94°C, 30 s, 57°C, 30 s, 72°C, 30 s, 30 cycles; (iii) 72°C, 1 min, 1 cycle; the PCR product was labeled with RedSafe^TM^ and separated by agarose gel electrophoresis to visualize the amplified fragments.

### Quantification of Aβ in the Cerebral Cortex of APP NL/F Mice

Aβ was extracted from the cerebral cortex of 3, 9, and 24 months APP NL-F mice. The mice were deeply sedated with an intraperitoneal (ip) injection of sodium pentobarbital (500 mg/Kg) and perfused with 0.9% sodium chloride. After perfusion, the brain was removed and the cortices were dissected and homogenized in 500 μl of homogenization buffer (0.5 M Tris HCl, 0.15 M NaCl Tween-20 0.05% 2 mM EDTA, pH 7.6) with protease inhibitors (cOmplete^TM^, Sigma-Aldrich). The sample was then ultracentrifuged at 200,000 *g* for 20 min at 4°C to separate the soluble and insoluble fraction. The supernatant (soluble fraction) was reserved for further analysis, and the pellet was resuspended in guanidinium chloride HCl (GndCl) 6 M, sonicated and stirred for 1 h at 23°C, to solubilize the insoluble Aβ. Then, it was ultracentrifuged at 200,000 *g* for 20 min at 4°C and the supernatant was collected (insoluble fraction).

The levels of Aβ38, Aβ40, and Aβ42 were measured by MSD (Meso Scale Discovery) multiplex immunoassay (Meso Scale Diagnostics, LLC) following the instructions of the manufacturer. Briefly, after blocking the wells with 150 μl of blocking buffer (0.1% casein in PBS) for 2 h at RT, they were washed 5 times with 200 μl of wash buffer (0.05% Tween-20 in PBS). Next, test samples or standard peptides (human Aβ38, Aβ40, and Aβ42 peptides) were diluted and prepared with the detection antibody (4G8) conjugated to an electrochemiluminescence label (SULFO-TAG^TM^) and loaded 50 μl in each well of the MSD multiplex plate. The plates were incubated at 4°C overnight and the next day they were washed 2 times with 150 μl of commercial development buffer (MSD T). Electrochemiluminescence was evaluated with the MSD Sector Imager 6000 apparatus.

### Determination of mRNA Levels in the Cerebral Cortex of APP NL/F Mice by Quantitative Reverse Transcription PCR (qRT-PCR)

RNA was extracted using Direct-zolTM RNA minipreps (Zymo Research ref.: R2052). RNA was quantified at 260 nm absorbance using a NanoDrop ND-100 (Thermo Fisher Scientific). The amount of mRNA present in mouse cortex or neurons in culture was assessed by qPCR. To this end, complementary DNA was synthesized from mRNA by reverse transcriptase PCR (RT-PCR) using a commercial kit (RevertAid H Minus First Strand cDNA Synthesis Kit, Thermo Fisher Scientific) under a 3-step PCR program (T^a^, duration): (i) 25°C, 5 min; (ii) 42°C, 60 min; (iii) 70°C, 5 min. Next, 5 ng of synthesized cDNA were used to perform the qPCR using a commercial kit (GoTaq® qPCR Master Mix, Promega). The oligonucleotides needed to amplify the region of interest were designed in regions of exons flanking long introns (intron-spanning), to avoid amplification of genomic DNA. The oligonucleotides used at a 0.5 μM final concentration for each gene are described in the following table.

**Table:** 

Gene	Sequence FWD	Sequence REV
*Bvra*	GGATATGTGTCCAGACGAGAAC	ATAGGCGACATCAACCTCTTG
*Ephx2*	GGAGAAGGTCACAGGGACAC	TTTGGATTGCATGGGACTG
*Foxo3a*	GTTTGGACCTTCGTCTCTGAA	GTAGTGTGACACGGAAGAGAAG
*Hsf1*	CCCTGAAGAGTGAGGACATAAA	GAGTCCATACACTCCTGTTTCC
*Ngb*	ACTGTCTCTCCTCTCCAGAAT	CAGGTACTCCTCCAATGAAGAC
*Pin 1*	GCAGAGGTCAGATGCAGAAA	TCTGTGCGCAGGATGATATG
*Grn*	GGGCATTTCTGCCATGATAAC	CAACAGTGACGTCCATCTCTAC
*Qpct*	CTGACAGCTGGGAATCTGAGT	TGAAGTCTCTGAATTCGTTGC
*Slc2a13*	GTCACCATCAACACCCTCTT	CATGTACCTCCATCCATCCTTC
*Tmem106b*	CTTGCCAAGGAACAGGAAGA	CACAGACGCCATCACATACA

The ABI PRISM 7900HT SDS (Applied Biosystems) equipment was used to perform the qPCR, in which a 3-step PCR protocol was programmed (T^a^, duration, number of cycles): (i) 95°C, 2 min, 1 cycle; (ii) 95°C, 3 s, 60°C, 30 s, 40 cycles; (iii) 95°C, 15 s, 60°C, 15 s and recovery with an increase of 7%, up to 95°C, 1 cycle. Ct (cycle threshold) was quantified as a relative measure of the concentration of PCR product. Fold changes were calculated with the ΔΔCt method (Livak and Schmittgen, 2001). The housekeeping gene PGK1 was used as an endogenous control. In addition, the fusion curves were analyzed to check the specificity of the reaction. All the experiments were performed in triplicate and qPCR data was analyzed with the SDS program (Applied Biosystems).

### Analysis of Protein Expression by Western Blot

Cortex of WT or APP NL/F mice were dissected for biochemical studies and homogenized in disruption buffer (Thermo Fisher Scientific) with protease inhibitors (cOmplete^TM^, Sigma-Aldrich), phosphatases (Sigma-Aldrich). For primary cultures, after the corresponding treatment they were washed with cold PBS and lysed in RIPA buffer (20 mM Tris–HCl, pH 7.5, 150 mM NaCl, 1 mM EDTA, 1 mM EGTA, 1% NP−40, 1% sodium deoxycholate, 0.1% SDS) with phosphatase inhibitors (Sigma-Aldrich) and proteases (cOmplete^TM^, Sigma-Aldrich). Proteins were prepared in Laemmli buffer (Tris–HCl 25 mM ph 6.8, sodium dodecyl sulfate (SDS) 1%, glycerol 3.5%, 2-mercaptoethanol 0.4% and bromophenol blue 0.04%) and separated by electrophoresis in polyacrylamide gels in the presence of SDS at constant voltage. Subsequently, they were transferred onto nitrocellulose membranes and after blocking with blocking solution [5% bovine serum albumin (BSA) in 0.1% Tween-20 in PBS (T-PBS)], membranes were incubated with the corresponding primary antibody diluted in blocking buffer overnight at 4°C. After washing the membranes with T-PBS, they were incubated with the relevant secondary antibodies coupled to horseradish peroxidase and diluted 1/5000 for 1 hr at RT. The proteins recognized by the antibodies were detected with luminol (Pierce^TM^ ECL Western Blotting Substrate, Thermo Fisher Scientific) and chemiluminescence was measured using a CCD camera (Amersham Imager 680). A given protein of interest was quantified by using the FIJI image-processing software to measure the average pixel intensity of the band corresponding to the protein in the digital image and was normalized with respect to the values obtained for the control protein GAPDH.

### Determination of Protein Concentration

The concentration of proteins present in the homogenates was determined by means of the BCA assay (Pierce^TM^ BCA Protein Assay kit, Thermo Fisher Scientific), following the indications of the commercial kit.

### LDH Toxicity Assay

The toxicity of the *in vitro* treatments was assessed by the release of the enzyme lactate dehydrogenase (LDH) to the extracellular medium. At the end of the corresponding treatment, 500 μl of the culture medium was collected and used to perform a colorimetric assay with a commercial kit (Pierce LDH Cytotoxicity Assay Kit, Thermo Fisher Scientific). The enzymatic reaction in the presence of LDH produces formazan, whose absorbance peak is 490 nm. The percentage of cytotoxicity of each treatment was calculated considering as 100% those cells treated with lysis buffer.

### Culture of Primary Cortical Neurons

The cerebral cortices from mouse embryos (E18) were dissected in HBSS medium (Hanks Buffer Salt Solution Ca2 + and Mg2 + free, Thermo Fisher Scientific) and digested with trypsin (Thermo Fisher Scientific) at 0.012% for 15 min at 37°C. The tissue was then disintegrated and resuspended in plating medium [Minimum Essential Media (Thermo Fisher Scientific) with 20% glucose and 10% horse serum (Invitrogen)]. The cells were seeded in the same medium on culture plates with coverslips pre-coated overnight at 37°C with 0.5 mg/ml poly-D-lysine (Sigma-Aldrich) at a density of 9,000 cells/cm2 for immunofluorescence studies or culture plates previously treated overnight at 37°C with 0.1 mg/ml poly-L-lysine (Sigma at a density of 10,000 cells/cm2 for biochemical analysis. After 3 h plating medium was replaced by Neurobasal medium (Thermo Fisher Scientific) supplemented with B27 (Thermo Fisher Scientific) and Glutamax (Thermo Fisher Scientific). Neurons were maintained at 37°C and 5% CO_2_ for the necessary time. Half of the medium was replaced by fresh medium once a week and Glutamax was removed on the 7th day *in vitro* (DIV 7).

### Treatment of Primary Cortical Neurons

#### Treatment of Primary Neurons With Synthetic Aβ42

Synthetic human Aβ42 (Anaspec) was resuspended in dimethyl sulfoxide (DMSO) at a concentration of 5 mM, and sonicated to ensure a homogeneous solution of monomers. In order to obtain oligomers, the peptide was diluted to 100 μM in DMEM and after vortexing, it was incubated overnight at 4°C. The state of aggregation of the peptide was verified by transmission electron microscopy. Due to the fact that B27 supplement contains antioxidants in its formulation, and antioxidants are known to inhibit Aβ-mediated toxicity (Ono et al., 2006; Manczak et al., 2010; Giordano et al., 2014; Rajasekhar et al., 2020), we replaced the media by Minimal Essential Medium (MEM) containing antioxidants-free N2 supplement, which has been previously used to study Aβ toxicity (Bateman et al., 2007; Uchida et al., 2011; Ungureanu et al., 2016). On DIV 8, the B27-supplemented medium was progressively replaced (1/4 of the media each day for 3 days, the 4th day being totally replaced) with MEM media supplemented with N2 (Gibco; Life Technologies Co.) and without GlutaMAX, and subsequently treated with oligomeric Aβ to study dendritic arborization (100 nM), mitochondrial respiration (10 μM) or Ngb expression (5 μM), at 8 DIV, 10 DIV or 14 DIV, respectively.

#### Treatment of Primary Neurons With H_2_O_2_

Prior to the treatment, the medium was progressively replaced (see above paragraph) with medium supplemented with N2 to eliminate antioxidants present in B27 supplement that could mask the action of H_2_O_2_. The H_2_O_2_ solutions were prepared fresh, making the corresponding dilutions in water and in such a way that the volume of treatment did not exceed 10% of the total volume of the well. Neurons were treated at 10 DIV with 10 μM H_2_O_2_ to assess mitochondrial respiration, or at 8 DIV with 1 and 10 μM to study Ngb expression.

### Determination of ROS Levels by DHR on Histological Sections of the Brain of hAPP NL/F Mice

The presence of ROS in the cortex of the mice was evaluated using dihydrorhodamine (DHR) (Thermo Fisher Scientific), a pigment that upon oxidation in the presence of ROS emits green fluorescence. After extracting the brain and making coronal sections of 350 μm thickness, the slices were kept for 1 h at 32°C with artificial cerebrospinal fluid (aCSF) bubbled with a mixture of 5% CO_2_ and 95% O2. Subsequently, they were incubated for 10 min with 10 μM DHR in aCSF at RT at darkness. After 2 washes of 15 min with PBS, the slices were fixed for 2 h with 4% PFA. Non-specific unions were blocked for 30 min with blocking buffer (2% BSA and 0.5% Triton X-100 in PBS). The cell nuclei were labeled with DAPI 1: 5000 in PBS for 15 min. The slices were mounted with Mowiol on a slide for observation under a confocal microscope (DAPI: λ = 405 nm, DHR: λ = 488 nm). The signal intensity in the green channel was evaluated after extracting the signal from the lipofuscin granules, which are autofluorescent at all wavelengths.

### Analysis of Dendritic Complexity

In order to study the complexity of the dendritic tree, dendrites were immunostained with anti-tGFP for neurons of animals injected with virus or anti-MAP2 for primary neurons infected in culture. In both cases, confocal images of isolated neurons at several planes (z stack) were taken with an overlap of 40%. The 8-bit images were processed with the Simple Neurite Tracer program of the FIJI software. Subsequently, the morphological complexity was studied by means of the Sholl analysis, in which the number of neurites crossing each of the imaginary circles of increasing radius (10 μm) that are drawn around the soma is determined. A graph is obtained in which the number of intersections from the cellular soma to the periphery is represented. The number of primary dendrites (projecting directly from the cell soma) and secondary or higher dendrites (projecting from a dendrite), as well as the number of bifurcation points were also quantified.

### Generation of Lentiviral Vectors

All the necessary procedures to produce lentiviruses were carried out in a culture room with a P2 biosecurity level. Second-generation lentiviral particles were created in order to introduce and integrate the mentioned plasmids in the neuronal nucleus in a stable and long-lasting manner. For this purpose, HEK 293-T cell line were transfected with a packaging plasmid (pCMV delta R 8.2, Addgene), a shell plasmid VSV-G (pMD2.G, Addgene) coding for vesicular stomatitis virus glycoproteins, and the following plasmids of interest: for the Ngb silencing experiment, a silencing plasmid (sh-Ngb) (Origene, TL503454a) or a scrambled (Scr) (Origene, TR30021) were used, while for the Ngb overexpression experiment, a plasmid containing the sequence for Ngb expression (Origene, NM_021257) was used. The transfection was carried out when cells reached 80% confluence by mixing the 3 plasmids prepared in OptiMEM (Thermo Fisher Scientific) and using polyethyleneimine (PEI, Sigma) in a PEI: DNA 1: 1 ratio. After 12 h, the medium was replaced by DMEM with 2% FBS and the cells were maintained at 37°C for an additional 48 h. After this time, the medium was collected and centrifuged at 900 *g* for 15 min at 4°C to remove cell debris and filtered with a 0.22 nm pore filter (Millipore). Subsequently the supernatant was ultracentrifuged at 60,000 *g* for 2 h at 4°C. The pellet corresponding to the lentivirus was resuspended in sterile PBS and frozen in single-use aliquots that were maintained at −80°C.

The concentration of lentiviruses, expressed in Transducing Units (TU)/ml, was estimated by flow cytometry. For this, HEK 293T cells were infected with increasing concentrations of virus and the percentage of infected cells (positive for tGFP) after 48 h was obtained. The 3 lentivirus preparations showed high titers, between 10^8^ and 10^9^ TU/ml. Neuronal cultures were infected at 6 DIV with a MOI (multiplicity of infection) of 5. To do this, on the day of infection, the amount of lentivirus needed was resuspended in 200 μl of conditioned medium and added to the culture of neurons dropwise. The next day, the medium was completely replaced by B27-supplemented Neurobasal medium.

### Immunofluorescence Analysis

#### Immunofluorescence of Neurons in Culture

Primary neurons on coverslips were washed 3 times with PBS and fixed with 4% PFA for 15 min at RT. Next cells were blocked and permeabilized with blocking solution (2% BSA and 0.1% Triton X-100 in PBS) for 1 h at RT and subsequently incubated overnight at 4°C with the corresponding primary antibody. After 3 washes with PBS, coverslips were incubated with secondary antibodies conjugated with fluorophores (1: 5000 in PBS) for 1 h at RT. Subsequently they were washed with PBS and the cell nuclei were labeled with DAPI (1: 5000 in PBS). Slices were mounted with Mowiol for observation under a confocal microscope.

#### Immunofluorescence of Brain Tissue

The left hemisphere of the brain of the mice, or the entire brain in the case of animals injected with lentivirus, was fixed by immersion in 4% PFA and 0.2 M sucrose in PBS overnight at 4°C. They were then cryoprotected with 30% sucrose in PBS, after which 40 μm thick sagittal sections were made with a vibratome (Leica VT1200 S). Sections were collected and maintained at −20°C in a cryoprotective solution (30% ethylene glycol and 26% glycerol in 0.1 M phosphate buffer (PB) pH 7).

Brain sections were permeabilized and blocked with blocking solution (2% BSA and 0.5% Triton X-100 in PBS) for 1 h at RT and subsequently incubated with the corresponding primary antibody overnight at 4°C. The next day and after performing 3 washes with PBS, the sections were incubated with secondary antibodies conjugated with fluorophores (dilution 1: 500 in blocking solution) for 1 h at RT. Then, they were washed with PBS and the nuclei were labeled with DAPI (1: 5000 in PBS) for 15 min. Sections were mounted with Mowiol on slides for analysis with a confocal microscope. Negative controls were included for each immunofluorescence reaction in order to check the specificity of the primary antibody. For this, sections were incubated only with the secondary antibody.

### Detection of Amyloid Plaques

The deposition of β-amyloid was detected by the fluorescent pigment thioflavin S (Th-S) (Merck), whose peak of excitation and maximum emission are 430 and 550 nm, respectively. The fixed brain sections were incubated for 15 min with 0.1% Th-S in 50% diluted ethanol, after which they were washed 2 times with 50% ethanol and subsequently with distilled water. The images were acquired with an inverted fluorescence microscope Axiovert 200 (Zeiss) coupled to a sCMOS camera (Excitation 461–488 nm, Emission 499–530 nm).

### Study of Mitochondrial Activity

Mitochondrial activity was assessed by 3- (4,5-dimethylthiazol-2-yl) -2,5-diphenyltetrazolium bromide (MTT) (Sigma). For this experiment, 10,000 neurons per well were plated in a 96-well plate and at 6 DIV they were infected with a control lentivirus or a lentivirus expressing Ngb. After the corresponding treatments at 10 DIV, the medium was replaced by 100 μl of 0.5% MTT diluted in N2-supplemented medium. The cells were kept at 37°C for 4 h to allow formation of formazan crystals, which were subsequently dissolved by adding 50 μl of DMSO. After 15 min at 37°C, the absorbance at 560 nm was read.

### LTP and LTD Experiments

Field excitatory postsynaptic potentials (fEPSPs) were recorded in the stratum radiatum of CA1 on hippocampal slices. The recording chamber was perfused with artificial CSF (aCSF) (120 mM NaCl, 2.5 mM KCl, 1 mM NaH2PO4, 11 mM glucose) with 0.1 mM picrotoxin and in bubbling with 5% CO_2_ and 95% O2. Bipolar stimulation electrodes were placed between the collaterals of Schaffer and the glass of the recording electrodes (1–2 MΩ, covered with aCSF) at a distance of 200 μm, in the direction of projection of the fibers. The depth of the electrode was adjusted to achieve a maximum synaptic response size in the given CA1 region. Records were made at 0.066 Hz with stimuli of 50 μs in duration, and a stable baseline of 20–30 min was acquired before experimental manipulation. An input-output curve was generated and a stimulus was established that would produce a maximum response of 50 or 30% of the maximum response for long-term depression (LTD) and long-term potentiation (LTP), respectively. The LTD was induced by low frequency stimulation of Schaffer collateral fibers (900 pulses at 1 Hz). A theta-burst stimulation protocol (TBS) was used to induce LTP: 4 series (at 20 s intervals) of 10 bursts (at 5 Hz) of 4 pulses (at 100 Hz).

### Water Morris Test

Before beginning the experiment, for 2 days the mice were introduced into the pool with a visible platform and their capacity to swim correctly was checked. In the learning phase, the mice were introduced into the pool facing the wall from 4 different positions each day, determined randomly. Mice were allowed to swim until they found the platform, and the time taken by each mouse was recorded. Four sessions per day were performed, with 1 min of rest between them, until the mice learned to find the platform (4 days).

On the 5th day, the test was performed to assess the memory of each subject. The platform was removed from the pool and the mouse was allowed to swim for 90 s. In this case, the time that the mouse spent in the quadrant where the platform was located (Q1) and in the opposite quadrant (Q3) was evaluated. From the 6th day, the inverse learning test was performed to evaluate learning dependent on the prefrontal cortex and working memory. For this, a hidden platform was reintroduced, but this time in Q3, so that the mice had to learn the new position of the platform. Four sessions per day were carried out for 4 days and the time it took for the subject to learn the new position of the platform was evaluated.

### Fear Conditioning Test

The hippocampal and amygdala-dependent memory was assessed using the fear conditioning test. In this test the mice receive an unconditioned stimulus (tone of 3 KHz of frequency and 80 dB of intensity) and a conditioned aversive stimulus (electrical discharges in the legs of 0.8 mA of intensity). The experiment takes place in a box with electrified metal floor and covered in red methacrylate to prevent the animal from seeing the outside, but allowing the experimenter to assess the movements of the mouse throughout the experiment.

On day 1, a mouse was introduced into the box and allowed to habituate to the environment for 3 min. After this time, 3 sessions of electric shocks of 2 s duration each one were applied and each of them preceded by a tone of 20 s. During this session, mice learn to associate the context and tone with the electric shocks.

On day 2, the same mouse was reintroduced in the same compartment for 5 min. The subjects capable of associating the context with the electric discharges show a behavior of total immobilization, whose duration was assessed as an index of memory associated with fear.

During day 3, the memory associated with the amygdala is evaluated, for which the context is completely changed (light conditions, smell, etc.) and the same tone is emitted as the first day. After allowing the mouse to get used to the new context for 2 min, the tone was emitted for 5 min. The immobilization time in this case is interpreted as the ability to associate the tone with the electric shocks, and measures a type of memory that is related to the amygdala.

The last day it was found that all the mice had the same sensitivity in the legs, for which the minimum intensity at which the subjects show signs of pain (vocalizations and jumps) was assessed.

### Stereotactic Injection

To study the effect of Ngb silencing *in vivo*, a lentivirus expressing an shRNA against Ngb (Ngb shLenti) was injected into the somatosensory cortex of 16-month-old APP NL/F mice, using the following coordinates: anteroposterior (Bregma): + 0.11; mediolateral: ± 0.15; dorsoventral: −0.18. We injected the shRNA lentivirus in one hemisphere and the control lentivirus expressing and scrambled shRNA (scr shLenti) in the contralateral hemisphere of the same mouse.

The mice were kept under deep sedation by inhalation of anesthesia (isoflurane) throughout the procedure. The loss of body temperature was controlled with the aid of a thermal blanket and the ocular dryness was prevented by ocular moisturizing cream.

The mouse head was fixed to the stereotactic apparatus by means of buccal and auricular fixations, and two holes were made in the skull with the help of a microdrill and a 1 mm diameter drill. Using a Hamilton syringe (Series 700 87930, Hamilton) with a 30G gauge needle (7762-03, Hamilton), 1 μl of the corresponding lentivirus was injected into each hemisphere at a rate of 0.2 μl/min.

At the end of the surgery, the incision in the scalp was sutured with surgical thread and local anesthetic cream was applied. The mouse was reanimated on an electric blanket and 200 μl of PBS ip was injected to prevent dehydration. Also, they were monitored for a week, with analgesic (Dalsy) in the drinking water. All procedures were carried out in an animal room with a P2 biosecurity level.

After 2 months, the mice were perfused and their brain cut into sections, as described above. With the help of an Axiovert 200 (Zeiss) fluorescence microscope, the sections were selected with infected cells for further study and analysis.

### Determination of NGB Levels in Plasma of Patients

Plasma samples were obtained from the Biobank of the Alzheimer Research Centre of the Queen Sofia Foundation. Plasma of donors was drawn in the first visit (V1) and the fifth visit, 5 years later (V5), and who did not show a cognitive decline between V5 and V1 (Stable group; Mean age: 75 years, *n* = 14), or with signs of mild cognitive impairment (MCI) at V5 versus V1 (Converters group; Mean age: 74.85 years; *n* = 7). Human blood samples were extracted, processed and stored at CIEN Foundation (Madrid, Spain) following the Vienna Protocol (Institute of Neurology, University of Vienna, May 2001. Modified proposal blood collection WHO 080501) essentially as previously described (Olazarán et al., 2015; Kenny et al., 2019). Briefly, after approval of the Local Ethics Committee and signing informed consent, blood samples were drawn in the presence of citrate and centrifuged at 2,280 × *g* for 5 min followed by separation of the plasma fraction and additional centrifugation for 10 min at 16,000×*g* to obtain the platelet-free plasma sample, which was stored at −80°C until use. The whole process was performed within 1 h of procurement.

After thawing, plasma was centrifuged at 1000 *g* at 4°C for 15 min and the supernatant was used to determine NGB levels with a commercial ELISA kit for the detection of human neuroglobin (Cloud-Clone Corp.) following the instructions of the manufacturers. The optimal dilution of plasmas to determine NGB levels was assessed in a pilot test. Briefly, plasma samples were diluted in 1X PBS and a standard curve was set up with 7 points: 500 pg/ml, 250 pg/ml, 125 pg/ml, 62,5 pg/ml 31.2 pg/ml, 15.6 pg/ml, 7.8 pg/ml. 100 μL of sample or standard were loaded onto the corresponding well of the ELISA plate and were incubated for 1 h at 37°C. After the incubation, 100 μL of Detection Reagent A provided by the manufacturer and the plate was incubated for 1 h at 37°C. After 3 times washes with Washing Buffer, 100 μL of Detection Reagent B was added per well and the plate was incubated for 30 min at 37°C (both buffers were provided by the manufacturer). The plate was washed 5 times with washing buffer, and 90 μL of Substrate Solution was added per well. After 20 min, the reaction was stopped with 50 μL of Stop Solution and absorbance was read at 450 nm in a spectrophotometer.

### Statistical Analysis

Statistical analyses of the data were performed with the SPSS (IBM) program. To analyze the effect of factors of two levels, the normality of the sampling distribution was checked by means of the Shapiro-Wilk test and the means were compared by means of the two-way Student *t*-test. In the case of factors of more than two levels, the one-way ANOVA followed by the Tukey *post hoc* test was used to compare the means between the different groups. To study the effect of 2 factors on a studied variable, a two-way ANOVA was performed to study the effect of each of the factors, as well as the possible interaction between them, and a Tukey *post hoc* analysis was followed to compare the means of the different groups. Values of P less than 0.05 were considered significant.

### Ethics Approval and Consent to Participate

All the experimental procedures with laboratory animals were carried out in accordance with the European directive transposed into the Spanish legal system in RD 53/2013, of 1 February, which establishes the basic rules applicable for the protection of animals used in experimentation and other scientific purposes.

The procedures for obtaining of plasma samples were approved by the Ethics Committee of Research and Animal Welfare of the Carlos III Health Institute. Donors signed an informed consent form and data was protected according the Spanish Law on Protection of Personal Data (15/1999).

## Results

### Absence of Alzheimer Signs in Old hAPP NL/F Mice

**FIGURE 1:**
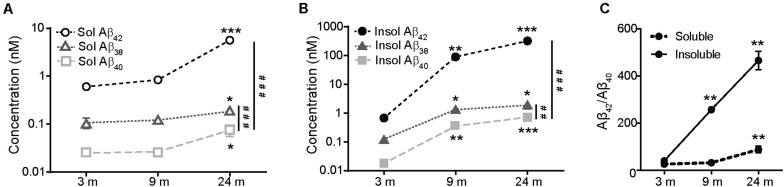
The levels of Aβ species increase in the cerebral cortex of APP NL/F during aging. **(A)** Quantification using the MSD-Vplex assay of the Aβ species present in the soluble extracts from the cerebral cortex of 3, 9, and 24 months-old APP NL/F mice. Aβ levels at 9 and 24 months were compared to 3 months (*n* = 4 mice per age and genotype; *p < 0.05, ***p < 0.001 vs. 3 months; ###*p* < 0.001 vs. Aβ 40). **(B)** Quantification using the MSD-Vplex assay of the Aβ species present in the insoluble extracts from the cerebral cortex of 3, 9, and 24 months-old APP NL/F mice. Aβ levels at 9 and 24 months were compared to 3 months (*n* = 4 mice per age and genotype; *p < 0.05, **p < 0.01, ***p < 0.001 vs. 3 months; ##*p* < 0.01, ###*p* < 0.001 vs. Aβ 40). **(C)** Changes of the pathologically relevant Aβ42/Aβ40 ratio over aging were plotted for insoluble and soluble Aβ by using the raw data shown in panels **(A,B)**. Aβ42/Aβ40 ratio at 9 and 24 months was compared to 3 months (*n* = 4 mice per age and genotype; **p < 0.01). Statistical significance was analyzed by one-way ANOVA followed by Tukey *post hoc* analysis.

Analysis of the concentration of different human Aβ species (Aβ38, Aβ40, and Aβ42) in the cerebral cortex of hAPP NL/F mice of various ages revealed a characteristic age-associated increase in both the soluble (Figure 1A) and the insoluble (aggregated peptide) (Figure 1B) fractions, already evident in 3-month-old mice. When we compared the increment of the soluble and insoluble forms of the different Aβ peptides over age, the highest increment corresponded to Aβ42, the most toxic form of the species analyzed (El-Agnaf et al., 2000; Yan and Wang, 2006), giving rise to the increase with age of the pathologically relevant Aβ42/Aβ40 ratio (Figure 1C).

Despite the high levels of Aβ and the increase in the Aβ42/40 ratio, the old hAPP NL/F mice, we could not detect major pathological changes in these mice. We were not able to detect, even in 24-month-old mice, microgliosis (Supplementary Figures S1A-C), pro-inflammatory (Supplementary Figures S1D,E) or anti-inflammatory (Supplementary Figures S1F,G) reaction. We also did not detect significant changes in proteasomal function (Supplementary Figure S2A), nor in protein stress response (Supplementary Figure S2B), which are found elevated in AD patients (Lukiw, 2004). The autophagosomal pathway was also not altered, reflected by similar levels of LC3 and P62 (Supplementary Figures S2C,D), nor was it the chaperone-mediated autophagy, as shown by the unaltered levels of LAMP2A along time (Supplementary Figure S2E). Also, no differences were observed in terms of activation of pro-apoptotic (Supplementary Figure S2F) or canonical pro-survival Akt activity (Supplementary Figures S2G,H) pathways. We did, however, detect an increase in the number of reactive astrocytes compared to age-matched WT mice (Supplementary Figure S3A) and an increase in the levels of tau phosphorylation at the pathogenic epitope Pair Helical Filament-1 (PHF1) (pSer396/404) (Supplementary Figure S3B).

**FIGURE 2:**
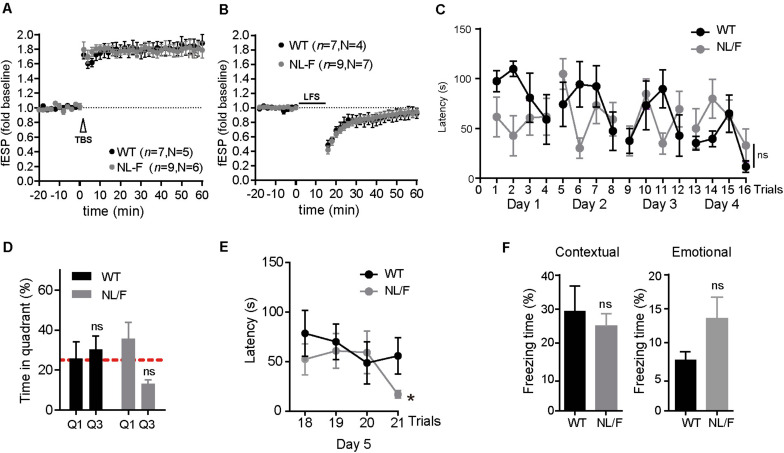
Synaptic plasticity, learning and memory are not affected in old APP NL/F mice. **(A,B)** LTP induction by TBS **(A)** and LTD by LFS **(B)** on hippocampal slices of WT and hAPP NL/F mice. The number n of slices used of N animals of each genotype is defined. The graphs show the mean ± SEM, two-way Student t, (ns: not significant, vs. WT). **(C)** Graphic representation of the latency time to the platform in APP NL/F and WT mice during the learning phase of the Morris water maze. Statistical significance was analyzed by T test of repeated samples. **(D)** Analysis of the percentage of swimming time in the target quadrant (Q1) and the opposite quadrant (Q3) on the day of the test (*n* = 5 WT mice and 7 NL/F mice). The graphs show the mean ± SEM. Statistical significance was analyzed by two-way T test. (ns: not significant vs. WT). **(E)** Reversal learning curve, in which the submerged platform has changed position (*n* = 5 WT mice and 7 NL/F mice). The graphs show the mean ± SEM. Statistical significance was analyzed by two-way T test. (ns: not significant) (*p < 0.05, vs. WT). **(F)** Graphic representation of the freezing time percentage of hAPP NL/F and WT mice during the exposure to the context related to the aversive stimulus (left) or to the tone (right) (*n* = 5 WT mice and 8 NL/F mice). The mean ± SEM is represented. Statistical significance was analyzed by *t* test of two ways (ns: not significant, vs. WT).

Given that excess of Aβ is associated with defects in synaptic transmission (Walsh et al., 2002; Li et al., 2009; Dinamarca et al., 2012), we carried out experiments to evaluate the hAPP NL/F mice at the electrophysiological and behavioral levels. For this, we performed both LTP (Long Term Potentiation) and LTD (Long Term Depression) recordings in hippocampal slices from old hAPP NLF and also Morris Water Maze and the Fear Conditioning tests. A strong LTP response was induced upon stimulation both in hippocampal slices from WT and hAPP NL/F mice (Figure 2A). Regarding the LTD response, it was initially induced but did not remain stable a long time, regardless of the genotype (Figure 2B). Similarly, we did not observe differences in memory and learning performance between WT and hAPP NL/F mice. As measured by the Morris Water Maze, both showed similar learning curves during training (Figure 2C), although strikingly, did not spend more time exploring the target quadrant (Q1) during the probe day, when the platform is removed (Figure 2D). When the platform position was changed, both WT and hAPP NL/F mice were able to learn the new position, the latter reaching the platform even faster than WT mice during the last trial (Figure 2E). Confirming these results, the Fear Conditioning test showed no differences in contextual (hippocampus-associated) memory or emotional (amygdala-dependent) memory (Figure 2F). Although these results may seem surprising given the high levels of toxic forms of amyloid peptide, similar results were observed in a previous work on the same hAPP NL-F mice (Masuda et al., 2016).

### Aβ42 Oligomers Trigger Neuroglobin Expression via Oxidative Stress

**FIGURE 3:**
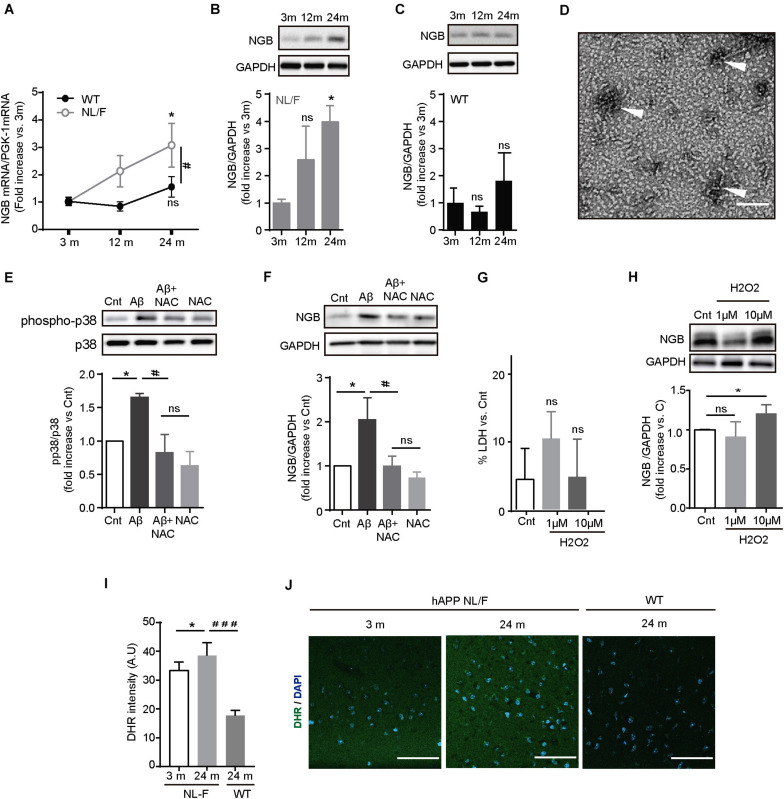
Ngb expression is enhanced in the cerebral cortex of old APP NL/F mice. **(A)** Ngb mRNA levels quantified in the cerebral cortex by qRT-PCR increase over aging only in hAPP NL/F mice. (*n* = 4 mice per genotype; *p < 0.05 vs. 3 months, #*p* < 0.05 vs. WT). **(B,C)** Ngb protein levels determined in the cerebral cortex by western blot increase in old hAPP NL/F **(B)** mice but not in WT **(C)** (of note: antibody specificity was tested by shRNA knockdown and by over-expression: Figure 1 for Reviewer 1). Plots show quantification of western blot experiments (*n* = 4 mice per age; *p < 0.05, ns: not significant, vs. 3 months). **(D)** Electron microscopy image of Aβ42 (scale bar = 100 nm). In order to obtain Aβ42 oligomers, synthetic human Aβ42 monomers were diluted in DMEM and incubated overnight at 4°C. Oligomers are pointed out by arrowheads. **(E)** Western blot showing the activation in 14 DIV primary neurons of p38 (phospho Tyr 180/Thr 182) after 24 h treatment with 5 μM Aβ42 oligomers [prepared like in Panel **(D)**] and its decrease with pre-treatment with 2 mM NAC (*n* = 6; bars represent the mean ± SEM; *p < 0.05, #*p* < 0.05, ns:not significant). **(F)** 5 μM Aβ42 oligomers trigger the expression of Ngb in primary cortical neurons and it is reverted by 2 mM NAC. A plot with the quantification of Ngb for the different conditions is shown (*n* = 6; *p < 0.05, #*p* < 0.05, ns: not significant). **(G)** Plot showing the toxicity of H_2_O_2_ treatment in cortical neurons determined by LDH release (*n* = 4; *p < 0.05 vs. vehicle (Cnt), ns: not significant). **(H)** 10 μM H_2_O_2_ treatment induces the expression of Ngb protein in cortical neurons determined by western blot. The plot shows the quantification of 4 independent western blot experiments (*p < 0.05, ns: not significant). **(I)** Analysis of the ROS labeling with DHR in cortical slices of hAPP NL/F mice, and WT mice (*n* = 12 slices from 3 mice of old WT and hAPP NL/F and 5 slices from 2 mice of young hAPP NL/F; *p < 0.05, ###*p* < 0.001). **(J)** Representative confocal images of hAPP NL/F mice of 3 and 24 months, and WT mice of 24 months, labeled against ROS with DHR. Scale bar: 50 μm. Statistical significance was analyzed by two-way (panel **A**) or one-way [panels **(B,C)** and **(E–I)**] ANOVA, without interaction, followed by Tukey *post hoc* analysis. Bars represent the mean ± SEM. (3 m, 12 m, 24 m means 3 months, 12 months, and 24 months, respectively).

As shown in the above series of results, even in the presence of high levels of toxic forms of Aβ over a long period of time, we could not find broad pathology in the cortex of this mouse model of AD. Hence, we envisioned that these mice put to work very robust defense mechanisms against the toxic effects of Aβ, perhaps similar to those operating in individuals without cognitive deficits but with a high amyloid load. To identify these mechanisms in these mice, we analyzed the expression levels of selected toxic (Supplementary Figures S4A,B) and survival (Supplementary Figures S4A,C) genes whose levels of expression were found altered in the brains of individuals affected by AD. Of the 9 genes studied, only Neuroglobin (Ngb) mRNA levels increased with aging in the hAPP NL/F mice but not in WT mice (Figure 3A). The increase in Ngb was also observed at the protein level only in the hAPP NL/F mice, whereas in the WT mice remained stable a long time (Figures 3B,C).

Ngb is an oxygen transporter and NO scavenger hemoprotein expressed in the brain and retina that protects cells from death after ischemia and reperfusion after damage (Burmester et al., 2000; Raychaudhuri et al., 2010; Tiso et al., 2011). Furthermore, Ngb increases in conditions of oxidative stress in different models (Li et al., 2008a, 2010; Watanabe et al., 2012; Amri et al., 2017). Given that Aβ also produces oxidative stress (Smith et al., 2007; Streltsov et al., 2008; Butterfield and Boyd-Kimball, 2019), and that our mice produce high amounts of Aβ (see Figures 1A–C), we studied next if the Aβ is able to increase the expression of Ngb. To test this possibility, we exposed mouse cortical neurons from wild type mice maintained in culture for 10 days to 5 μM Aβ42 oligomers for 24 h. The phosphorylation status of P38 MAPK, which is enhanced upon Aβ exposure via a mechanism involving oxidative stress, was increased after treating neurons with Aβ oligomers (Figure 3D) and this effect was reduced if neurons were pre-treated with 2 mM of the anti-oxidant N-acetylcysteine (NAC), thus proving the efficacy of the protocol used. Importantly, Aβ42 oligomers increased Ngb protein levels and this was reversed by NAC (Figure 3E), pointing to ROS as the mediator of the effect. Consistent with this assumption, treating primary neurons with a non-toxic concentration of H_2_O_2_ (Figure 3F) increased Ngb expression (Figure 3G). We also observed high levels of ROS in the cortex of hAPP NL/F mice, which would also indicate that the age-associated increase in Ngb in these mice is strongly associated with increased oxidative stress, likely linked to the presence of elevated levels of Aβ (Figures 3H–J).

### Ngb Protects Neurons Against Aβ-Induced Mitochondrial Stress

**FIGURE 4:**
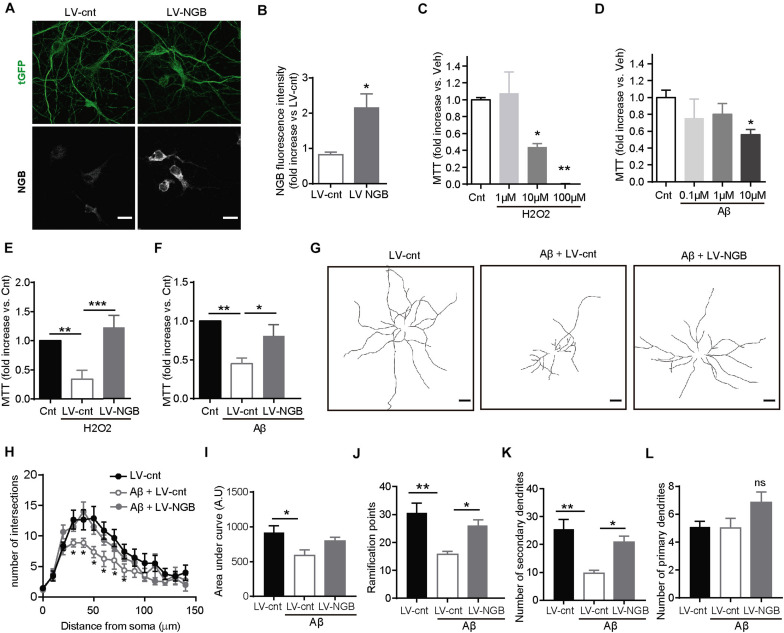
Overexpression of Ngb rescues H_2_O_2_- and Aβ-induced mitochondrial dysfunction and loss of dendritic complexity. **(A)** Representative confocal images of neurons infected with control lentivirus (LV-Cnt) or lentivirus overexpressing Ngb (LV-NGB) both expressing tGFP, and immunostained for Ngb and tGFP. Scale bar: 20 μm. **(B)** Graph showing the intensity for Ngb calculated from confocal images like the one shown in Panel **(A)** (*n* = 10 neurons from 3 independent cultures; *p < 0.05). **(C)** Curve showing mitochondrial activity measured by MTT assay after exposing primary cortical neurons to different concentrations of H_2_O_2_ [*n* = 3; *p < 0.05, **p < 0.01 vs. vehicle (Cnt)]. **(D)** Curve showing mitochondrial activity measured by MTT assay after exposing primary cortical neurons to different concentrations of oligomeric Aβ42 [*n* = 3; *p < 0.05, **p < 0.01 vs. vehicle (Cnt)]. **(E,F)** Decrease in mitochondrial activity of cortical neurons determined by reduction of MTT and induced by 10 μM H_2_O_2_ **(E)** or 10 μM Aβ42 oligomers **(F)** is rescued by overexpressing Ngb (LV-NGB) (*n* = 8; *p < 0.05, **p < 0.01, ***p < 0.001). **(G)** Representative traces of the dendritic arborization of 14 DIV neurons in culture infected with a control lentivirus (LV-cnt) or a lentivirus overexpressing Ngb (LV-NGB), and treated or not with 100 nM Aβ42 oligomers for 24 h (Scale bar: 20 μm). **(H)** Sholl analysis to determine the dendritic complexity of neurons treated or not with 100 nM Aβ42 oligomers and infected with a control lentivirus (LV-cnt) or a lentivirus to overexpress Ngb (LV-NGB) (*n* = 15 neurons from 3 independent cultures of each group; *p < 0.05). **(I)** Graph representing the area under the curve calculated from Panel **(H)** (*p < 0.05). **(J–L)** Dendritic bifurcations **(J)** and higher-order dendritic branches **(K)** are less abundant in primary neurons treated with 100 nM Aβ42 oligomers compared to non-treated cortical neurons, while no statistical differences are observed for primary branches **(L)**. These effects are reverted by overexpression of Ngb (LV-NGB) (*p < 0.05, **p < 0.01). The graphs show the mean SEM. Statistical significance was analyzed by one-way ANOVA followed by Tukey’s *post hoc* analysis, except for panel B, which was analyzed by Statistical significance was analyzed by two-tailed *t* test.

Aβ oligomers produce neuronal toxicity through, among many other mechanisms, affecting mitochondrial function (Lustbader et al., 2004; Manczak et al., 2010). In this direction, it has been proposed that Ngb protects against cell death by decreasing nitric oxide (NO) formation and preventing cytochrome C-induced caspase 9 activation (Raychaudhuri et al., 2010; Tiso et al., 2011). To determine whether or not increased levels of Ngb protect neurons against Aβ-toxicity, primary cortical neurons were transduced with empty lentiviral particles (LV-cnt) as a negative control or lentiviral particles containing the DNA sequence for Ngb (LV-NGB) (Figures 4A,B). 4 days post-transduction, 10 μM H_2_O_2_ or Aβ oligomers, at concentrations known to be toxic (Figures 4C,D), were added to the media and the levels of reduced MTT by mitochondria evaluated. While Aβ oligomers and H_2_O_2_ treatment decreased the reduction of MTT in control-infected neurons (Figures 4E,F), those infected with LV-NGB had levels of reduced MTT similar to neurons that had not been treated either with Aβ or hydrogen peroxide (Figures 4E,F), thus confirming the anti-stress, protective role of this protein.

### Ngb Protects Against Aβ Oligomers-Induced Dendritic Shrinkage

Aβ is widely known to be itself an important source of ROS (reviewed in Butterfield and Boyd-Kimball, 2019). In turn, accumulated ROS can have deleterious consequences on the function of brain cells, including the perturbation of the neurons’ dendritic architecture (Landino et al., 2004; Fukui et al., 2011). Mechanistically, alterations in the stability of microtubule are considered responsible for the architectural defects observed in a highly oxidized environment (Landino et al., 2004; Fukui et al., 2011). Thus, Aβ, perhaps via oxidative stress (but not only), would affect microtubule organization and dynamics and consequently neuronal architecture. In support of this possibility, recent works have shown that changes in microtubules and dendritic simplification are among the earliest manifestation of Aβ toxicity (Wu et al., 2010; Deng et al., 2019). More importantly, dendritic tree simplification is a typical feature of early AD, both *in vivo* in animal models of the disease and in the human brain (Golovyashkina et al., 2015). Therefore, in view of our results showing that the over-expression of Ngb reduces the oxidative stress induced by Aβ peptide, we investigated if the higher levels of Ngb in the brains of our hAPP NL/F mice could have prevented the appearance of architectural defects produced by this peptide, and consequently the initiation of the AD phenotype. To test this possibility, we first analyzed the effect of ectopic Ngb on Aβ-mediated dendritic architecture alterations *in vitro* analyzed by the number of dendritic crossings of circles centers at the soma and with increasing radius (Figures 4G,H). Consistent with published work, 100 nM Aβ oligomers reduced the complexity of the dendritic tree of primary cortical neurons in culture (Figures 4G–I). Dendritic bifurcations (Figure 4J) and second- or higher-order dendrites (Figure 4K) were less abundant in primary cortical neurons treated with Aβ oligomers compared to non-treated neurons. These results are in agreement with published findings that Aβ triggers the pruning of dendrites progressively from the synapses inward, affecting spines and higher-order branches without impacting the number of primary dendrites. This, however, did not happen for primary dendritic branches and in neurons overexpressing Ngb (Figures 4J–L).

**FIGURE 5:**
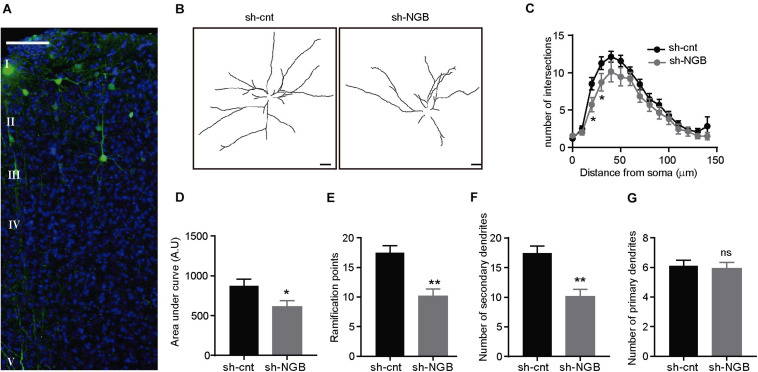
Ngb downregulation reduces the dendritic complexity of cortical pyramidal neurons in old APP NL/F mice. **(A)** Confocal image representative of the area infected with a lentivirus expressing an shRNA against Ngb and turboGFP (tGFP) (sh-Ngb). Nuclei are stained with DAPI and cortical layers are indicated. **(B)** Representative traces of the dendritic arborization of cortical neurons of the layer III infected with a control lentivirus (sh-cnt) or sh-Ngb to downregulate Ngb. (Scale bar: 20 μm). **(C)** Sholl analysis to determine the dendritic complexity of neurons infected with a control (sh-cnt; *n* = 17 sh-cnt neurons in total analyzed from 4 mice) or silencing (sh-Ngb; *n* = 17 sh-Ngb neurons in total analyzed from 4 mice) lentivirus (*n* = 34 neurons in total from 8 mice *p < 0.05). Statistical significance was analyzed by one-way ANOVA followed by Tukey’s *post hoc* analysis. **(D)** Graph representing the area under the curve calculated from Panel C (*p < 0.05). The graph shows the mean ± SEM. Statistical significance was analyzed by *t* test. **(E–G)** Dendritic bifurcations **(E)** and higher-order dendritic branches **(F)** are less abundant in neurons infected with sh-Ngb, while no differences are observed for primary dendritic branches (**p < 0.01). The graphs show the mean ± SEM. Statistical significance was analyzed by one-way ANOVA followed by Tukey’s *post hoc* analysis **(C)** and *t* test **(D–G)**.

To determine if this role of protecting the integrity of the dendritic tree occurs *in vivo*, we injected a lentivirus expressing an shRNA against Ngb (LV-shRNA-Ngb) or a scrambled shRNA (LV-shRNA-cnt) (Supplementary Figures S5A,B) in the cortex of old hAPP NL/F mice (Figure 5). Mice were sacrificed 2 months post-injection and their brains were analyzed by immunofluorescence. Interestingly, downregulation of Ngb levels caused a reduction of dendritic complexity in the neurons of the injected area (Figure 5A) as revealed by Sholl analysis, not observed in LV-shRNA-cnt injected animals (Figures 5B–D). Similar to the results obtained *in vitro* after Aβ treatment, downregulation of Ngb resulted in fewer dendritic bifurcations (Figure 5E) and fewer second- or higher-order dendritic branches dendrites (Figure 5F), with no effect on primary branches (Figure 5G). On the other hand, Ngb downregulation did not result in more amyloid plaques (Supplementary Figure S5C) or neuronal loss (Supplementary Figure S6) near the injected area, suggesting that the upregulation of Ngb that constitutively occurs in the hAPP NL/F mice is interfering with Aβ toxicity. According to the observation that Ngb increases with age in the hAPP NL/F but not wild type mice, reduction of Ngb in the brain of old wild type mice had no consequences on dendritic tree architecture.

### Plasma Levels of NGB May Predict Conversion to Mild Cognitive Impairment

**FIGURE 6:**
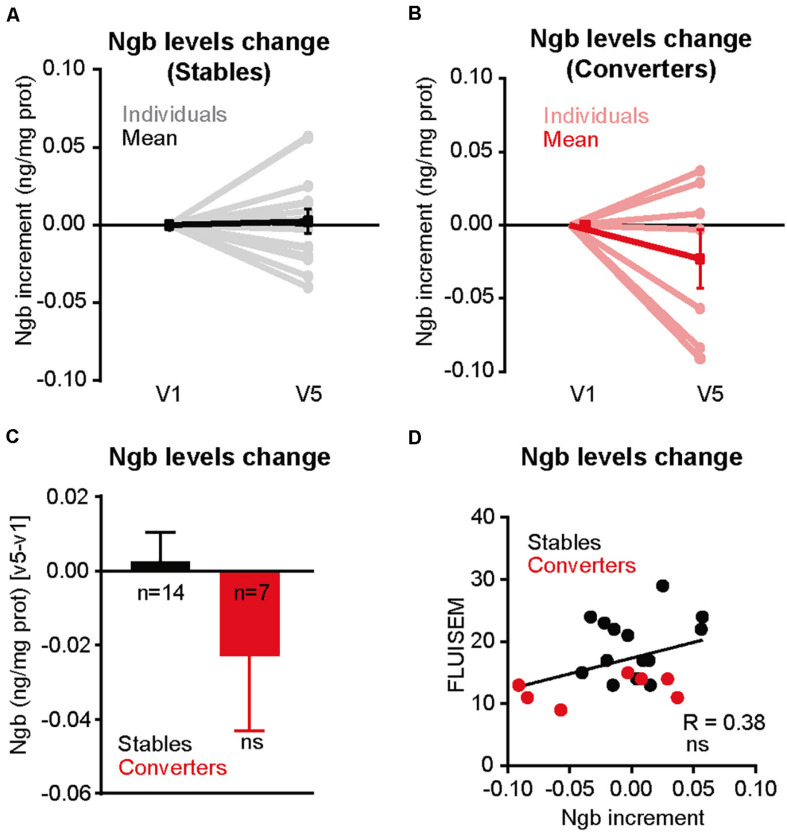
Cognitively altered patients show a decrease in Ngb plasma levels. **(A)** Representation of the differences in the levels of plasma neuroglobin, normalized by total protein, calculated from the blood of individuals (*n* = 14) extracted in the first visit (V1) and the fifth visit, 5 years later (V5), and who did not show a cognitive decline between V5 and V1 determined by several neurological and neuropsychological tests (Stable). The plasma Ngb levels obtained at V1 was set at 0 for comparative purposes. **(B)** Same representation as in A, but for individuals with signs of mild cognitive impairment (MCI) at V5 versus V1 (Converters). **(C)** Bar plot illustrating the differences in Ngb levels (normalized by total plasma proteins) between V5 versus V1, for Stable (*n* = 14) and Converters (*n* = 7) Individuals. Negative values indicate a decrease in absolute Ngb levels between V1 and V5. **(D)** Correlation between the amount of Ngb levels change (V5 versus V1) and the score obtained by the neuropsychological semantic fluency test FLUIsem. Subjects who score less than 15 in this test are considered to have mild dementia. Negative values in the *X* axis indicates a decrease in absolute Ngb levels between V1 and V5.

Our data supports a role of NGB in offering protection to neurons against Aβ-induced toxicity. Thus, people with lower levels of NGB in the brain may be at higher risk of cognitive impairment, especially in the elderly as a consequence of the higher levels of toxic Aβ species generated (Dewachter et al., 2000; Placanica et al., 2009; Guix et al., 2012; Janssen et al., 2016). We tested this possibility by analyzing blood NGB levels. We took this approach based on the previous demonstration that NGB levels were shown to peak up 72 h after an episode of acute ischemic stroke (Xue et al., 2017). With the aim of determining if lower plasma NGB levels are associated with a higher risk of developing dementia, we carried out a pilot longitudinal study to determine the NGB levels from plasma of healthy donors drawn the first visit (V1) and 5 years later (V5), when some of them had developed different degrees of cognitive impairment (see Table 1). Then we calculated the difference in plasma NGB levels between V5 and V1 and compared the degree of change between donors who stayed cognitively stable at V5 (Stables; *n* = 14) and donors who showed a mild cognitive impairment (MCI) at V5 (converters; *n* = 7) (Table 2). Interestingly, the highest drops in plasma NGB levels were detected in the group of the converters (Figures 6A,B). In average, the group of converters showed a tendency to decrease plasma NGB levels between V1 and V5, but it did not reach statistical significance (Figure 6C). On the other hand, when we compared the plasma NGB levels with the score obtained in different neuropsychological tests we found that subjects with a lower FLUIsem, a semantic fluidity test used to diagnose dementia in which a score below 15 is associated to mild dementia (Pena-Casanova et al., 2009), presented lower NGB levels (Table 1 and Figure 6D).

**TABLE 1:** Demographics and neuropsychological tests.

	Stable		Converters	
	*Visit 1*	*Visit 2*	*Visit 1*	*Visit 2*
Gender	7F/7M		5F/2M	
Age (mean ± SD)	75.07 ± 0.26		74.85 ± 4.29	
MMSE	28.57	28.43	26.85	26.43
FAQ	0.07	0.22	1.43	6.33
FLUIsem	18.72	19.36	14.43	12.43
CDR	0	0	0	0.5

*Demographics and scores obtained during the neuropsychological evaluation for the different tests and for the two groups analyzed (Stable and Converters). The neuropsychological evaluation in Visit 2 occurred 5 years after Visit 1.*

**TABLE 2:** Determination of neuroglobin (Ngb) levels in the plasma.

Sample ID	Visit 1 (V1)	Visit 5 (V5)	Visit 1 (V1)	Visit 5 (V5)	V5-V1
	Diagnose	Diagnose	Ngb [ng/mg	Ngb [ng/mg	Ngb [ng/mg
			protein]	protein]	protein]
0223	non-MCI	non-MCI	0.098	0.084	−0.014
0338	non-MCI	non-MCI	0.068	0.046	−0.022
0418	non-MCI	non-MCI	0.121	0.125	0.004
0688	non-MCI	non-MCI	0.076	0.132	0.056
0894	non-MCI	non-MCI	0.121	0.088	−0.033
0945	non-MCI	non-MCI	0.050	0.107	0.057
0012	non-MCI	non-MCI	0.079	0.039	−0.040
0111	non-MCI	non-MCI	0.051	0.060	0.009
0282	non-MCI	non-MCI	0.061	0.075	0.014
0562	non-MCI	non-MCI	0.060	0.041	−0.020
0742	non-MCI	non-MCI	0.074	0.089	0.015
0762	non-MCI	non-MCI	0.087	0.112	0.025
0888	non-MCI	non-MCI	0.087	0.071	−0.015
1102	non-MCI	non-MCI	0.045	0.042	−0.003
0090	non-MCI	MCI	0.121	0.064	−0.057
0270	non-MCI	MCI	0.154	0.070	−0.084
0308	non-MCI	MCI	0.084	0.081	−0.003
0611	non-MCI	MCI	0.165	0.074	−0.091
0770	non-MCI	MCI	0.035	0.063	0.029
0912	non-MCI	MCI	0.039	0.076	0.037
1177	non-MCI	MCI	0.047	0.054	0.008

*The table shows the Ngb levels (normalized by total protein) from plasma of healthy donors drawn the first visit (V1) and 5 years later (V5), when some of them had developed different degrees of cognitive impairment (MCI) (see Table 1). The difference in plasma Ngb levels between V5 and V1 (V5-V1) was calculated.*

## Discussion

Several works have demonstrated the protective capacity of Ngb in different brain toxicity contexts, including AD (Khan et al., 2007; Li et al., 2008b, 2010, 2016; Chen et al., 2012). However, no previous work has shown that this route of protection is *constitutively activated in vivo*, both in the mouse and human brain, in parallel to age and Aβ increase, and that this increase is required to *prevent the appearance* of one of the earliest signs of disease: neuronal architectural deficiencies. Herein, in the demonstration that Ngb upregulation is a physiological protective response, lies the originality of our work.

A number of observations support the above conclusion: (1) the levels of Ngb increase with age and in parallel to the increase in brain Aβ in the hAPP NL/F mice, not in wild type; (2) Ngb is activated by Aβ, in an oxidative stress mediated manner, which is consistent with the role of Ngb as an anti-oxidant (in fact, we found that the hAPP NL/F/G knock-in mice, which present earlier Aβ deposition in comparison to the hAPP NL/F mice, show a statistically significant increase of Ngb as early as at 11–13 months of age (Supplementary Figure S7), (3) its increase in wild type neurons prevents the cytoarchitectural defects induced by Aβ, (4) its downregulation *in vivo*, in the hAPP NL/F mice leads to the appearance of dendritic tree simplification. Although it would seem logical to attempt to confirm the relevance of Ngb in the prevention of AD phenotype by crossing the hAPP NL/F mice with Ngb KO mice (Hundahl et al., 2012), these mice do not present abnormalities in anatomy, neuronal loss, body composition or behavior, implying that Ngb physiological (protective) roles can be efficiently compensated when missing constitutively by any of the multiple survival mechanisms present in neurons (Hundahl et al., 2011, 2012). In support to this hypothesis, it was unexpectedly found that Ngb-null mice showed a reduced infarct size after inducing an experimental stroke (Raida et al., 2012). Thus, the acute reduction of Ngb via shRNA that we have done in this work gives a valid approximation to the possible function of this protein in conditions of supra-physiological stressors, such as the high levels of toxic forms of Aβ. However, crossing hAPP NL-F mice with inducible KO Ngb mice could certainly strengthen (or weaken) our interpretation.

Given that the simplification of the dendritic tree is one of the earliest events of Aβ toxicity (Yamada et al., 1988; Flood, 1991; Hanks and Flood, 1991; Tsai et al., 2004; Grutzendler et al., 2007), we find reasonable to propose that the increase in Ngb is in the first line of defense against the harmful effects of Aβ. Although the increase in Ngb may also counteract the toxicity derived from the high levels of PHF1 observed in old hAPP NL/F mice, we cannot exclude that the increase in PHF1 in the old hAPP NL-F is itself a sign of resistance to neuronal death rather than of degeneration (Li et al., 2007; Yang and Wang, 2018). From mechanistic data provided in previous studies on the anti-apoptotic role of Ngb, we presume the protection of architectural integrity of dendrites by Ngb in a high Aβ context is indirect, through the inhibition of mitochondrial damage-induced ROS/NO production (Raychaudhuri et al., 2010; Tiso et al., 2011) which are known to affect the stability of microtubules (Landino et al., 2004; Fukui et al., 2011). On the other hand, the protection against the occurrence of architectural deficiencies by Ngb is important so that no major damage by Aβ occurs. As a matter of fact, previous works have shown that overexpression of Ngb prevents Aβ-induced cell death (Khan et al., 2007; Li et al., 2008b, 2016) and attenuates memory impairment and amyloid plaque formation (Li et al., 2016), all of them late events in the course of AD. However, the observation of a drop in NGB plasma levels in individuals who have progressed from cognitive normal to MCI -which we assume reflects a drop in brain levels-, together with the mice data would imply that NGB is a defensive mechanism during the early phases of Alzheimer’s disease. In support of this possibility are also the results showing that a polymorphism in Ngb that decreases the expression of the protein increases the risk of developing AD (Szymanski et al., 2010).

The acceptance of the scenario described above would be compatible with the idea that elevating the NGB levels during the early clinical phases of the disease may prevent the progression of AD symptoms. That this is a reasonable therapeutic approach comes from the recent demonstration that systemic administration of a recombinant NGB, with higher oxygen binding affinity and higher NO reduction capacity, is effective to prevent carbon monoxide poisoning (Azarov et al., 2016).

## Data Availability Statement

The raw data supporting the conclusions of this article will be made available by the authors, without undue reservation.

## Ethics Statement

The studies involving human participants were reviewed and approved by Ethics Committee of the Carlos III Institute of Health. The patients/participants provided their written informed consent to participate in this study. The animal study was reviewed and approved by CBMSO’s Ethical Committee.

## Author Contributions

CD designed the overall approach, coordinated the study, and drafted the manuscript. SV, FG, and CD prepared the manuscript. SV did most of the biological studies and statistical analysis of the data. FG contributed to the design of the different experiments and executed a number of them. IP-P prepared neuronal primary cultures and handled the experimental animal model. AF-G revised the manuscript and provided intellectual inputs. TS and TCS provided the knock-in mice. JD did the electrophysiological studies. MS and LC-G determined the Aß levels. MC and MM provided the human plasma samples. MM provided critical analysis of the experiments and the results. All authors read and approved the final manuscript.

## Conflict of Interest

The authors declare that the research was conducted in the absence of any commercial or financial relationships that could be construed as a potential conflict of interest.
