# Supplementary material for: Prodromal Alzheimer’s Disease: Constitutive Upregulation of Neuroglobin Prevents the Initiation of Alzheimer’s Pathology
Source: Front Neurosci. 2020 Dec 3;14:562581. doi: 10.3389/fnins.2020.562581 (PMC7744294; doi:10.3389/fnins.2020.562581)
Supplement: Supplementary file 1 [file Data_Sheet_1.pdf]

SUPPLEMENTARY FIGURE S1

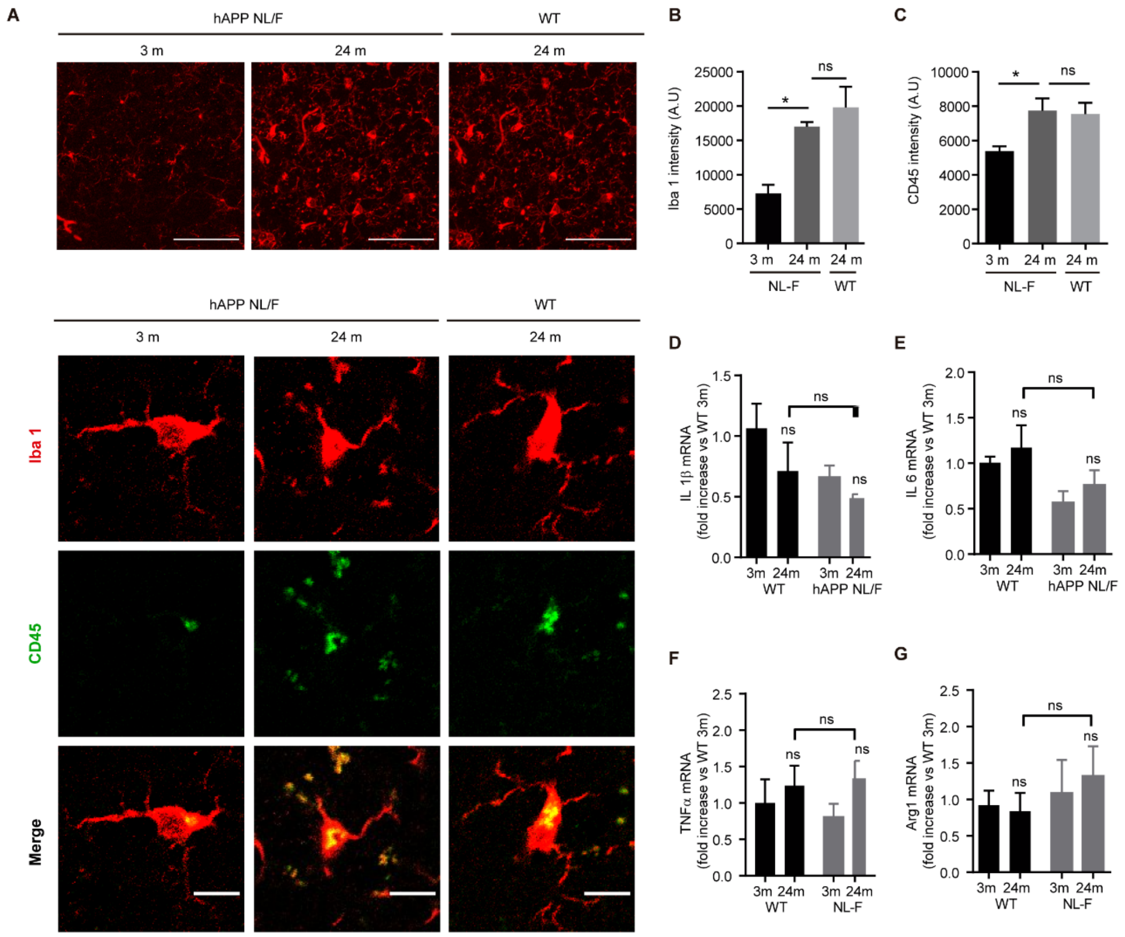

Supplementary Figure S1. Old APP NL/F and WT mice show the same level of microglial activation in the cerebral cortex.

(A) Representative confocal images showing immunostaining of cortical microglia with Iba 1 and double immunostaining of activated microglia with Iba 1 and CD45 in the cerebral cortex of hAPP NL/F mice of 3 and 24 months, and WT mice of 24 months. **(B)** Graph representing the intensity of Iba1 in the cerebral cortex of hAPP NL/F mice of 3 and 24 months, and WT mice of 24 months, quantified from confocal images like the one shown in Panel A (n=4 mice; * P <0.05; ns = not significant). **(C)** Graph representing the intensity of CD45 in the cerebral cortex of hAPP NL/F mice of 3 and 24 months, and WT mice of 24 months, quantified from confocal images like the one shown in Panel A (n=4 mice; * P <0.05; ns: not significant). **(D, E)** Levels of mRNA of the different pro-inflammatory cytokines quantified by qPCR in the cerebral cortex of hAPP NL/F and WT mice at 3 and 24 months of age (n = 4 mice; ns: not significant). **(F,G)** Levels of mRNA of the different anti-inflammatory cytokines quantified by qPCR in the cerebral cortex of hAPP NL/F and WT mice at 3 and 24 months of age (n = 4 mice; ns: not significant). The graphs show the mean ± SEM, one-way ANOVA followed by Tukey post hoc analysis

SUPPLEMENTARY FIGURE S2

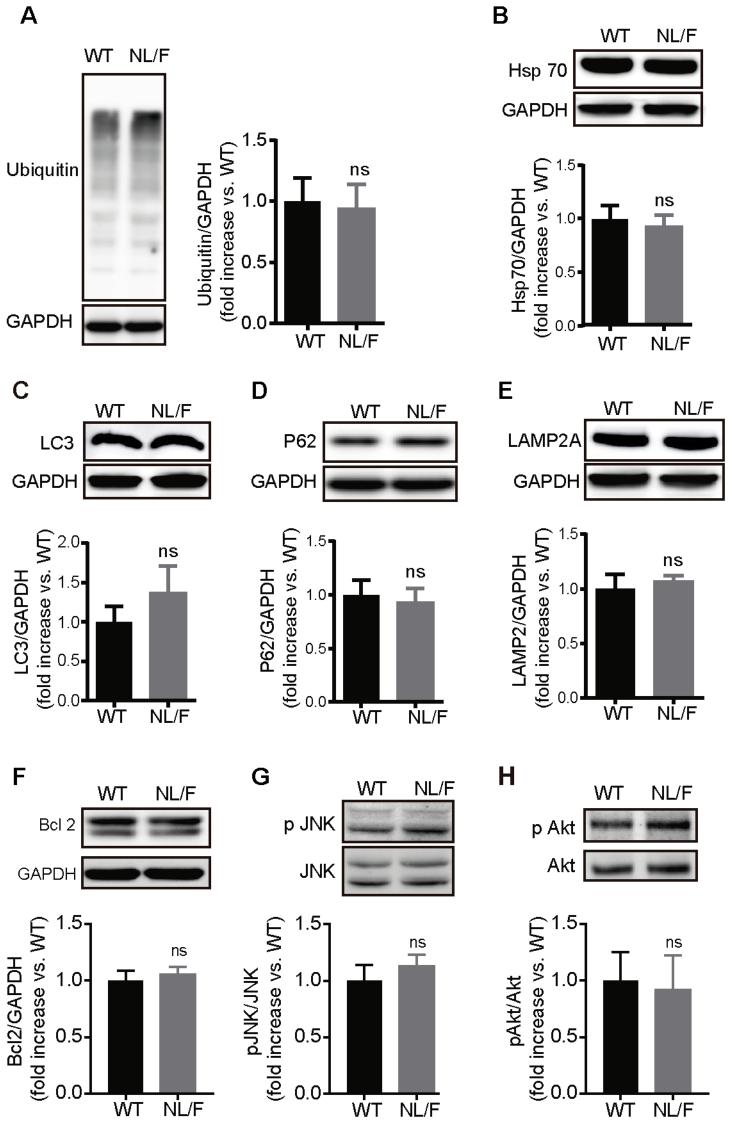

Supplementary Figure S2. Old APP NL/F mice do not show altered cellular proteostasis or stress. **(A)** Western blot analysis of protein ubiquitination levels in the cerebral cortex of WT and hAPP NL/F mice. GAPDH was used a control for protein loading. Quantification of the bands of western blot experiments as the one in this panel show no differences in the levels of ubiquitination between WT and hAPP-NL-F animals (n=3 mice of each group; ns: not significant). **(B)** Western blot analysis of the expression of the HSP70 chaperone in the cerebral cortex of WT and APP NL/F mice. GAPDH was used a control for protein loading. Quantification of the bands of western blot experiments as the one in this panel show no differences in the levels of HSP70 between WT and hAPP-NL-F animals (n=7 mice of each group; ns: not significant). **(C-E)** Western blot analysis of the levels of the proteins involved in the autophagy process LC3, p62 and LAMP2A in cortical extract of WT and APP NL/F mice. GAPDH was used a control for protein loading. Quantification of the bands on western blot experiment as the ones shown in these panels show no differences in the levels of LC3, P62 and LAMP2A between WT and hAPP NL/F animals (n=7 mice of each group for LC3 and LAMP2A; n=3 mice of each group for P62; ns: not significant). **(F)** Western blot analysis of the expression of the pro-apoptotic protein Bcl2 in the cerebral cortex of 24-month-old WT and APP NL/F mice. GAPDH was used a control for protein loading. Quantification of the bands on western blots as the one shown in this panel shows no differences in the levels of Bcl2 between WT and hAPP-NL-F animals (n=5 mice; ns: not significant). **(G)** Western blot analysis of the activation of the pro-survival protein JNK in the cerebral cortex of WT and hAPP NL/F mice. Phosphorylated JNK levels (phospho Thr 183/Tyr 185) were normalized to total JNK.

Quantification of the bands on western blot experiments as the one in this panel show no differences in the activation of JNK between WT and hAPP-NL/F animals (n=5 mice; ns: not significant). **(H)** Western blot analysis of the activation of the pro-survival protein Akt in the cerebral cortex of WT and hAPP NL/F mice. Phosphorylated Akt levels (phospho Ser473) were normalized to total Akt. Quantification of the bands on western blots as the one shown in this panel show no differences in the activation of Akt between WT and hAPP-NL-F animals (n=5 mice; ns: not significant). The graphs show the mean ± SEM. Statistical significance was analyzed by t test.

SUPPLEMENTARY FIGURE S3

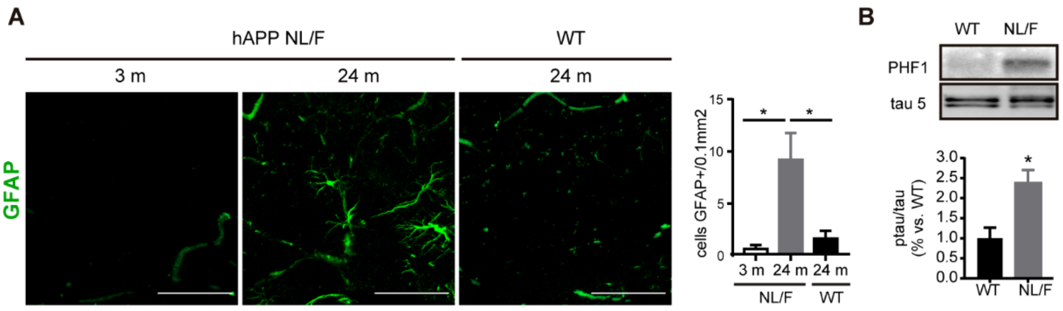

Supplementary Figure S3. **Old APP NL/F mice exhibit cortical astrocyte activation and tau phosphorylation.**

(A) Representative confocal images (left) and its quantification (right) of immunostaining with GFAP performed in the cortex of hAPP NL/F mice of 3 and 24 months, and WT mice of 24 months. The graph show the mean ± SEM. Statistical significance was analyzed by one-way ANOVA followed by Tukey post hoc analysis (n = 4 mice; *P <0.05). Scale bar: 50 µm. **(B)** Western blot analysis of the levels of phosphorylation of tau in the pathogenic epitope PHF 1 (phospho Ser 396 / Ser 404) in the cerebral cortex of WT and hAPP NL/F mice. PHF1 tau levels were normalized to total tau detected with the Tau 5 antibody. Quantification of the bands on western blots as the one shown in this panel show higher levels of PHF1 tau in the cortex of hAPP NL/F compared to WT mice. The graph show the mean ± SEM. Statistical significance was analyzed by t test (n=5 mice; *p<0.05).

SUPPLEMENTARY FIGURE S4

A

	Gene	Protein	Role
Toxicity	Foxo3a	Forkhead box O3	Oxidative stress
	Qpct	Glutaminyl-Peptide Cyclotransferase	Aβ aggregation
	Tmem106b	Transmembrane Protein 106B	Lysosome trafficking
Survival	Blvra	Biliverdin Reductase A	Insulin signaling
	Ephx2	Epoxide Hydrolase 2	Inflammation
	Grn	Granulin precursor	Lysosome trafficking
	Hsf-1	Heat shock factor 1	Protein folding
	Pin-1	Peptidyl-Prolyl Cis/Trans Isomerase) NIMA-Interacting 1	Protein folding

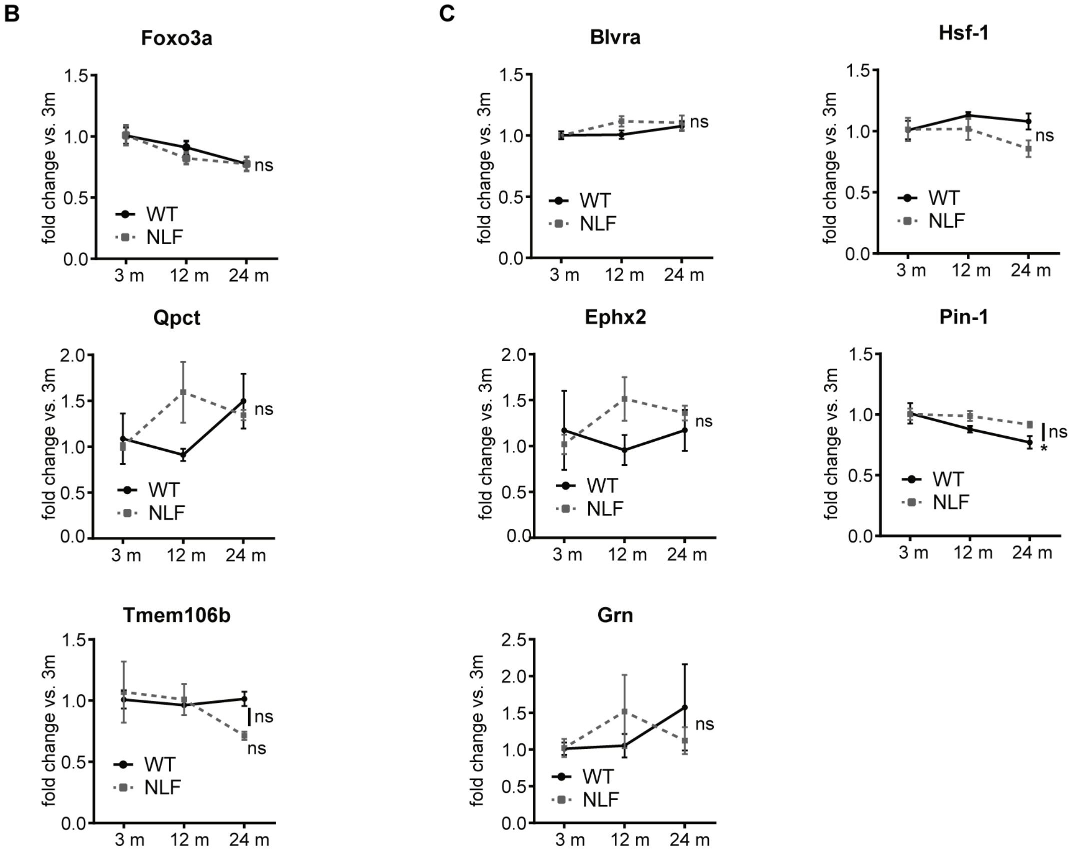

Supplementary Figure S4. **qRT-PCR analysis of toxicity and survival genes in the cortex of APP NL/F and WT mice.**

(A) Summary of the genes selected and grouped according to their function in toxicity or survival processes. **(B-C)** Analysis by qRT-PCR of genes related to toxicity (B) or survival (C) during AD in the cortex of WT and APP NL/F mice at 3, 12 or 24 months of age. Note that the expression of none the selected genes changes over time in hAPP NL/F as compared to WT mice (n = 4 mice of each genotype). The graphs show the mean ± SEM. Statistical significance was analyzed by two-way ANOVA (genotype, age), without interaction, followed by Tukey post hoc analysis (* P <0.05 vs. 3m, #P <0.05 vs. WT; ns: not significant).

SUPPLEMENTARY FIGURE S5

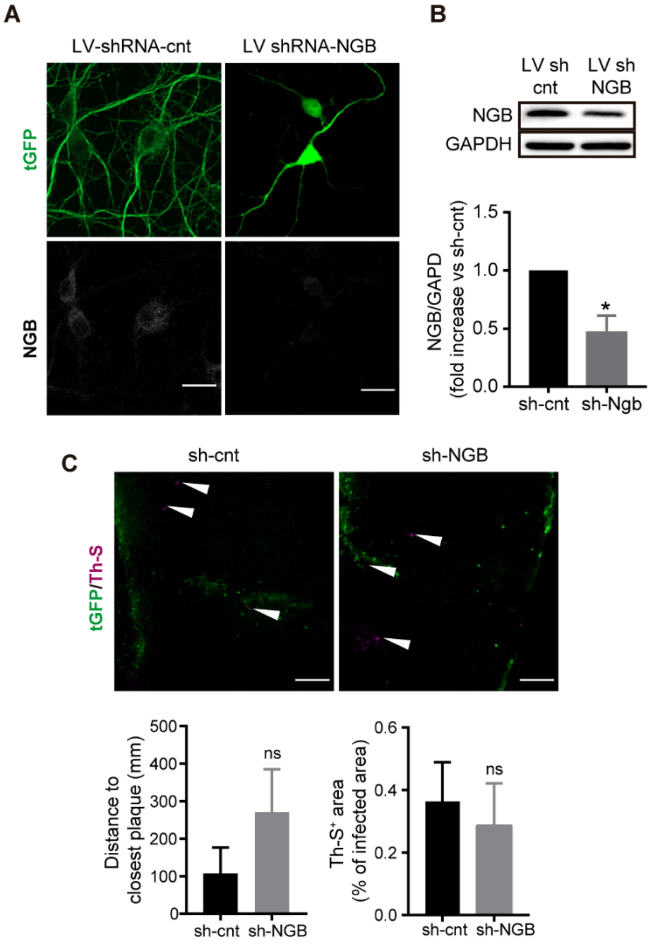

Supplementary Figure S5. NGB silencing in cortical neurons does not affect formation of amyloid plaques.

(A) Representative confocal images of primary cortical neurons infected with a control lentivirus expressing a scrambled shRNA (LV shRNA-cnt) or a lentivirus expressing an shRNA against NGB (LV shRNA-NGB). Both lentiviruses contain the tGFP reporter. Neurons were inmunostained for NGB and tGFP. Scale bar: 20 µm. **(B)** Western blot analysis of NGB expression in primary cortical neurons infected with LV shRNA-cnt and LV shRNA-NGB (n = 12 neurons from 3 independent cultures). The graph represents the mean ± SEM. Statistical significance was analyzed by two-way t test (* P <0.05 vs. Scr). **(C)** Representative confocal images of the area of infection in the cerebral cortex of 18-month old hAPP NL/F mice injected with a control lentivirus expressing a scrambled shRNA (sh-cnt) or a lentivirus expressing an shRNA against NGB (sh-NGB). Both lentiviruses contain the tGFP reporter. tGFP signal correspond to infected neurons and amyloid plaques are visualized by thioflavin staining (Th-S). Arrow heads indicate amyloid plaques (n = 4 mice). The graphs represents the distance of the closest amyloid plaque to a particular infected neuron (left) and the area covered by amyloid plaques (right). No differences were found in terms of amyloid plaques distribution or size between mice infected with the Scr or the sh-NGB lentiviruses. The graphs show the mean ± SEM. Statistical significance was analyzed by two-way t test (ns: not significant). Scale bar: 200 µm.

SUPPLEMENTARY FIGURE S6

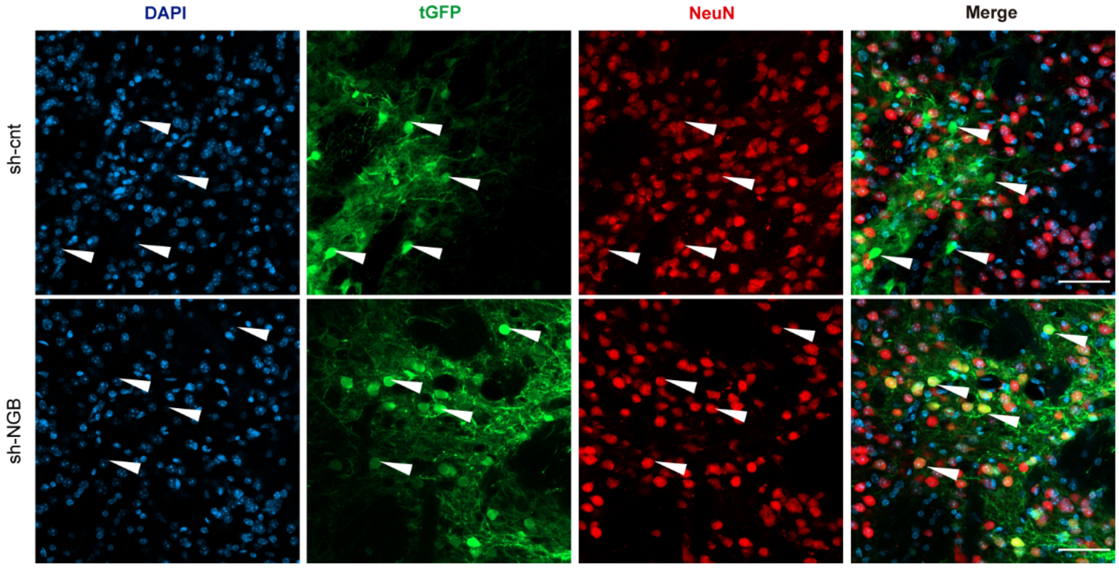

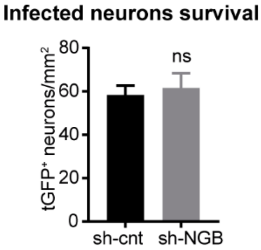

Supplementary Figure S6. Silencing of NGB in the cortex of old APP NL/F does not induce neuronal death.

Representative confocal images of the 18 -month-old hAPP NL/F cortex infected with a control lentivirus expressing a scrambled shRNA (sh-cnt) or a lentivirus expressing an shRNA against NGB (sh-NGB) (both lentiviruses contain the tGFP reporter; above) and the quantification of the number of neurons positive for tGFP and of total neurons in the area of infection (below). The arrowheads show examples of cells positive for NeuN and tGFP (n = 4 mice). The graph shows the mean ± SEM. Statistical significance was analyzed by two-way t test (ns: not significant). Scale bar: 50 μm.

SUPPLEMENTARY FIGURE S7

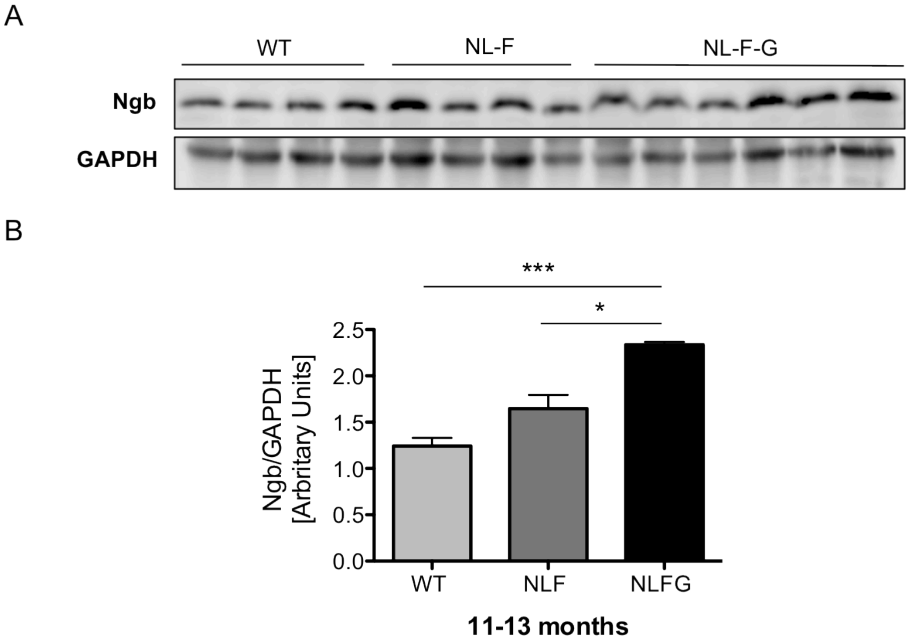

Supplementary Figure S7. APP NL-F-G mice show a strong up-regulation of Ngb in the cerebral cortex.

(**A**) Total lysates of the cerebral cortex of 11-13 months WT mice (n=4), APP NL-F knock-in mice (NLF; n=4) and APP NL-F-G knock-in mice (NLFG; n=6) were analyzed by western blot with an antibody against neuroglobin (Ngb) or GAPDH (loading control). **B**) Quantification of the western blot in Panel A. The graph represents the mean ± SEM. Statistical significance was analyzed by t test (* P <0.05; *** < 0.001). *(*Of note: quantification was done only with males, so to be comparable with a similar experiment carried out in h-NLF mice at 3, 12 and 24 months of age (see Figure 3). Preliminary results revealed a non-significant increase of Ngb in 13 month-old NL-F-G females*)
